# High prevalence of *Pentatrichomonas hominis* infection in gastrointestinal cancer patients

**DOI:** 10.1186/s13071-019-3684-4

**Published:** 2019-08-28

**Authors:** Nan Zhang, Hongbo Zhang, Yanhui Yu, Pengtao Gong, Jianhua Li, Ziyi Li, Ting Li, Zhanjie Cong, Chunying Tian, Xiaofeng Liu, Xiuyan Yu, Xichen Zhang

**Affiliations:** 1 The First Hospital, Key Laboratory of Zoonosis Research by Ministry of Education, Institute of Zoonosis, Jilin University, Changchun, 130021 China; 2 Key Laboratory of Zoonosis Research by Ministry of Education, Institute of Zoonosis, College of Veterinary Medicine, Jilin University, Changchun, 130062 China; 3 Clinical Lab, The Second Hospital, Jilin University, Changchun, 130021 China; 4 Jilin Cancer Hospital, Changchun, 130021 China

**Keywords:** *Pentatrichomonas hominis*, Gastrointestinal cancer, Colorectal cancer, Stomach cancer, Epidemiology

## Abstract

**Background:**

*Pentatrichomonas hominis* is a flagellated protozoan that inhabits the large intestine of humans. Although several protozoans have been proposed to have a role in cancer progression, little is known about the epidemiology of *P. hominis* infection in cancer patients.

**Methods:**

To determine the prevalence of *P. hominis* in patients with digestive system malignancies, we collected 195 and 142 fecal samples from gastrointestinal cancer patients and residents without any complaints related to the digestive system, respectively. Each sample was detected for the presence of *P. hominis* by nested PCR amplifying the internal transcribed spacer (ITS) region and partial *18S* rRNA gene.

**Results:**

A significantly higher prevalence of *P. hominis* was found in cancer patients than that in the control population (41.54 *vs* 9.15%, *χ*^2^ = 42.84, *df* = 1, *P *< 0.001), resulting in a 6.75-fold risk of gastrointestinal cancers (OR: 6.75, 95% CI: 3.55–12.83, *P *< 0.001). The highest prevalence of *P. hominis* infection was detected in small intestine cancer patients (60%, OR: 14.88, 95% CI: 0.82–4.58, *P* = 0.009) followed by liver (57.14%, *χ*^2^ = 10.82, *df* = 1, *P* = 0.001) and stomach cancer patients (45.1%, *χ*^2^ = 31.95, *df* = 1, *P *< 0.001). In addition, phylogenetic analysis provided some evidence supporting that human *P. hominis* infection might derive from animal sources.

**Conclusions:**

To our knowledge, this study is the first report presenting the high association between *P. hominis* and gastrointestinal cancers. Nevertheless, whether there is any possible pathological role of *P. hominis* infection in cancer patients needs to be further elucidated.

## Background

Cancer is a leading health burden worldwide, with an estimated 18.1 million new cancer cases and over nine million deaths in 2018 [1]. Gastrointestinal cancer, including colorectal, stomach, liver and esophagus cancers are the most commonly diagnosed malignancies, contributing to an incidence of over 23.8% [1, 2]. Among them, colorectal cancer is the third most commonly diagnosed cancer and the second leading cause of cancer-related deaths in the world, closely followed by stomach and liver cancer for mortality [1]. Although great efforts have been made into the investigation and treatment of cancer, it is still an unresolved health burden due to, at least partially, lack of a detailed understanding of its causative and influence factors.

In addition to genetic/epigenetic defects and environmental factors, pathogens also play a role in the induction and/or progression of cancer. Infectious agents, including viruses, bacteria and parasites, are estimated to account for about 20% of the global cancer incidence [3, 4]. Currently, several pathogens have been identified to be able to induce or contribute to human cancers [5–7]. Strikingly, many of them are closely related to gastrointestinal cancers. The well-known infectious agent of stomach cancer is *Helicobacter pylori* which can cause chronic gastritis [8, 9]. Hepatitis B and C viruses are the most important risk factors for hepatocellular carcinoma [10, 11]. In addition, the liver flukes *Opisthorchis viverrini* and *Clonorchis sinensis* can cause cholangiocarcinoma [12]. Nevertheless, the relationship between pathogens and cancers is still underestimated worldwide.

Emerging evidence has linked several protozoans to cancers [13–15]. *Trichomonas vaginalis*, a sexually transmitted pathogen, was reported to associate with cervical and prostate carcinoma [16, 17]. *Tritrichomonas musculis* can induce the release of the proinflammatory cytokine IL-18, which in turn contributes to the colorectal carcinoma in mice [18]. An epidemiological study in Uzbekistan indicated that *Blastocystis* sp. was highly associated with colorectal cancer (80%) [14]. Other studies also showed that *Blastocystis* sp. infection can facilitate the proliferation of colorectal cancer cells and downregulate the host immune cell response [19, 20]. Additionally, several epidemiological studies have indicated a high association between *Cryptosporidium parvum* infection and colon cancer, and have suggested that *C. parvum* could be a potential causative agent of this disease [21, 22].

*Pentatrichomonas hominis*, belonging to the Trichomonadidae, inhabits the digestive tract of several vertebrates such as humans, monkeys, pigs, dogs, cats and rats [23–27]. This species was originally considered a commensal protozoan of the digestive tract but has subsequently been identified as a potential zoonotic parasite and a causative agent of diarrhea [28–32]. *Pentatrichomonas hominis* has also been associated with irritable bowel syndrome, systemic lupus erythematosus and rheumatoid arthritis in humans [30, 33, 34]. Therefore, its impact on human health and disease remains unsettled. To investigate the *P. hominis* infection situation in gastrointestinal cancer patients, we conducted a case-control study in Jilin Province, China.

## Methods

### Collection of fecal samples

A total of 337 fecal samples were collected from the Jilin Cancer Hospital and the Second Hospital of Jilin University in China during January–July 2018. Among them, 195 specimens were submitted by inpatients with confirmed diagnosis of gastrointestinal cancers, including colorectal cancer (*n* = 116), stomach cancer (*n* = 51), esophageal cancer (*n* = 16), liver cancer (*n* = 7) and small intestine cancer (*n* = 5). The gastrointestinal cancer cases were determined by hospital imaging (B ultrasound; computed tomography, CT; nuclear magnetic resonance, NMR) and pathological diagnosis (gold standard). No patients in this study were found to use immunosuppressants in the hospital. Of the 195 patients, 125 were male and 70 were female with an average age of 59 (ranging from 31 to 87 years; Additional file 1: Table S1). One hundred and thirteen patients came from urban areas and 82 from rural areas. In contrast, 142 samples were collected from the control population (local residents who had no complaints relating to the gastrointestinal tract), including 71 males and 71 females with average age of 60 (ranging from 19 to 80 years; Additional file 2: Table S2). The control population who had no correlation with cancers were confirmed by health examination in the hospital. The fresh fecal samples were collected in individual containers, and were stored at − 20 °C until DNA extraction which was generally performed within 24 h. In order to avoid data deviation caused by potential cross-contamination of stool samples, the sample preparation area, PCR amplification area and sample analysis area were strictly distinguished, and an internal reference control of the sample was set up at the same time.

### DNA extraction and nested PCR analysis

DNA was extracted from each fecal specimen using a Fecal DNA Rapid Extraction Kit (TIANGEN, Beijing, China) following the manufacturer’s instructions. All DNA samples were detected for the presence of *P. hominis* by nested PCR amplifying the partial *18S* rRNA gene as previously described [27]. In addition, we performed TA cloning for each positive sample and amplified the ITS1/*5.8S* rRNA gene/ITS2 genomic region as previously described [35]. The genomic DNA extracted from *P. hominis* (ATCC 3000) and ddH_2_O were used as positive and negative controls, respectively. All DNA specimens were analyzed twice. The positive PCR products were purified and sequenced.

### Sequence analysis and phylogeny

The nucleotide sequences obtained were aligned with known sequences retrieved from the GenBank database on the BLAST website (https://blast.ncbi.nlm.nih.gov/Blast.cgi). All novel nucleotide sequences obtained in this study were deposited in the GenBank database under the accession numbers MK177542–MK177552, MN173974–MN173996 and MN189982. The phylogenetic tree was constructed using the maximum likelihood (ML) method under the Kimura 2-parameter model with bootstrap values out of 1000 replicates using MEGA v.7.0 (http://www.megasoftware.net/).

### Statistical analysis

All statistical analyses were performed in SPSS Statistics 20.0 software (IBM, Armonk, NY, USA). Pearson’s Chi-square test or Fisher’s exact test were used to estimate the statistical significance. All statistical tests were two-sided. Unconditional logistic regression analysis was used to determine the association of *P. hominis* infections with the risk of different cancers by calculating odds ratios (ORs) and 95% confidence intervals (CIs). All ORs were adjusted for both age and sex. *P *< 0.05 was considered to be statistically significant.

## Results

### High association of *P. hominis* infections with gastrointestinal cancers

**Fig. 1:**
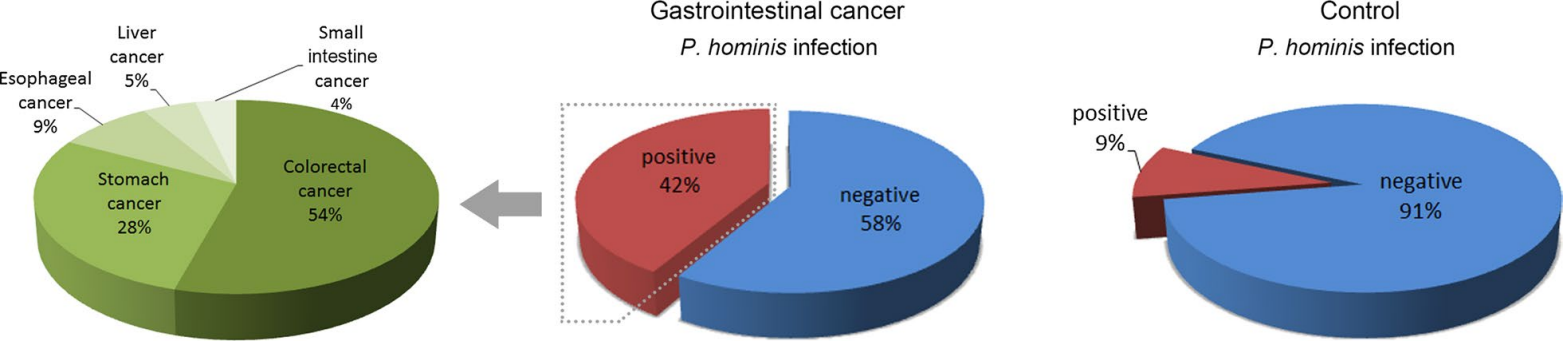
Pie graph of the proportion of *P. hominis* infections in gastrointestinal cancers (left and middle) and control population (right)

For the prevalence study, a total of 337 fecal samples were collected from 195 gastrointestinal cancer inpatients and 142 control population. Overall, the *P. hominis* infection rate in cancer patients was 41.54%, which was significantly higher than that in controls (9.15%, *χ*^2^ = 42.84, *df* = 1, *P *< 0.001; Fig. 1, Table 1). Intriguingly, *P. hominis* were found in all the studied cancer types, including colorectal cancer (37.93%, 44/116), stomach cancer (45.1%, 23/51), esophageal cancer (43.75%, 7/16), liver cancer (57.14%, 4/7) and small intestine cancer (60%, 3/5) (Table 1). Colorectal cancer accounted for 54%, stomach cancer accounted for 28%, while others accounted for 18% of *P. hominis* infections (Fig. 1). These results indicate a frequent occurrence of *P. hominis* in gastrointestinal cancers.

**Table 1:** Prevalence of *P. hominis* infection in the control population and gastrointestinal cancer patients

Group	No. examined	No. positive (%)	No. negative (%)	*χ*^2^/*df*/*P*-value	Logistic regression analysis		
					OR^a^	95% CI	*P*-value
Control	142	13 (9.15)	129 (90.85)	Reference	1	Reference	–
Gastrointestinal cancer	195	81 (41.54)	114 (58.46)	42.84/1/< 0.001	6.75	3.55–12.83	< 0.001
Cancer types							
Colorectal cancer	116	44 (37.93)	72 (62.07)	30.72/1/< 0.001	5.93	3.00–11.77	< 0.001
Stomach cancer	51	23 (45.1)	28 (54.9)	31.95/1/< 0.001	7.11	3.13–16.11	< 0.001
Esophageal cancer	16	7 (43.75)	9 (56.25)	12.59/1/< 0.001^b^	34.85	5.94–204.40	< 0.001
Liver cancer	7	4 (57.14)	3 (42.86)	10.82/1/0.001^b^	20.38	3.29–126.28	0.001
Small intestine cancer	5	3 (60)	2 (40)	–/–/0.009 (OR: 14.88; CI: 0.82–4.58)^c^	14.77	1.03–211.18	0.047

^a^Adjusted for age and sex

^b^Continuity correction

^c^Fisher’s exact test

The age of cancer patients ranged between 31–87 years. *Pentatrichomonas hominis* infections were found in all age groups, with the peak in 50–60 years (45.9%, 28/61; Table 2). In addition, the infection rate in males (44.8%, 56/125) was higher than that in females (35.71%, 25/70) (Table 2). A slightly more common infection was observed in cancer patients from urban areas (43.4%, 49/113) than that from rural areas (39.02%, 31/82) (Table 2). However, there was no statistically significant difference between any of these groups. A similar tendency of the infection rates was also observed in colorectal cancer and the control population (Table 2, Additional file 3: Table S3). Nevertheless, *P. hominis* infection in stomach cancer was significantly enriched in males compared to females (55.26 *vs* 15.38%, *χ*^2^ = 6.22, *df* = 1, *P* = 0.01; Additional file 4: Table S4).

**Table 2:** Prevalence of *P. hominis* infections in gastrointestinal cancer and colorectal cancer patients by selected characteristics

Group	Gastrointestinal cancer (*n* = 195)				Colorectal cancer (*n* = 116)			
	No. examined	No. positive (%)	No. negative (%)	*χ*^2^/*df*/*P*-value	No. examined	No. positive (%)	No. negative (%)	*χ*^2^/*df*/*P*-value
Age (years)								
≤ 50	44	16 (36.36)	28 (63.64)	0.97/2/0.62	28	10 (35.71)	18 (64.29)	0.94/2/0.62
51–60	61	28 (45.9)	33 (54.1)		31	14 (45.16)	17 (54.84)	
> 60	90	37 (41.11)	53 (58.89)		57	20 (35.09)	37 (64.91)	
Sex								
Male	125	56 (44.8)	69 (55.2)	1.53/1/0.22	67	25 (37.31)	42 (62.69)	0.03/1/0.87
Female	70	25 (35.71)	45 (64.29)		49	19 (38.78)	30 (61.22)	
Residence								
Urban	113	49 (43.4)	64 (56.6)	0.37/1/0.54	71	28 (39.44)	43 (60.56)	0.33/1/0.57
Rural	82	32 (39.02)	50 (60.98)		44	15 (34.09)	29 (65.91)	

To further determine the association between *P. hominis* infections and gastrointestinal cancers, unconditional logistic regression analyses were conducted. Overall, *P. hominis* infections resulted in a 6.75-fold (95% CI: 3.55–12.83) risk of gastrointestinal cancers after adjustment for age and sex. In detail, *P. hominis* infections were associated with a 5.93-fold (95% CI: 3.00–11.77), 7.11-fold (95% CI: 3.13–16.11), 34.85-fold (95% CI: 5.94–204.40), 20.38-fold (95% CI: 3.29–126.28) and 14.77-fold (95% CI: 1.03–211.18) risk of colorectal, stomach, esophageal, liver and small intestine cancer, respectively. These results suggest a high association of *P. hominis* with gastrointestinal cancers.

### Molecular characterization of *P. hominis* polymorphism

**Fig. 2:**
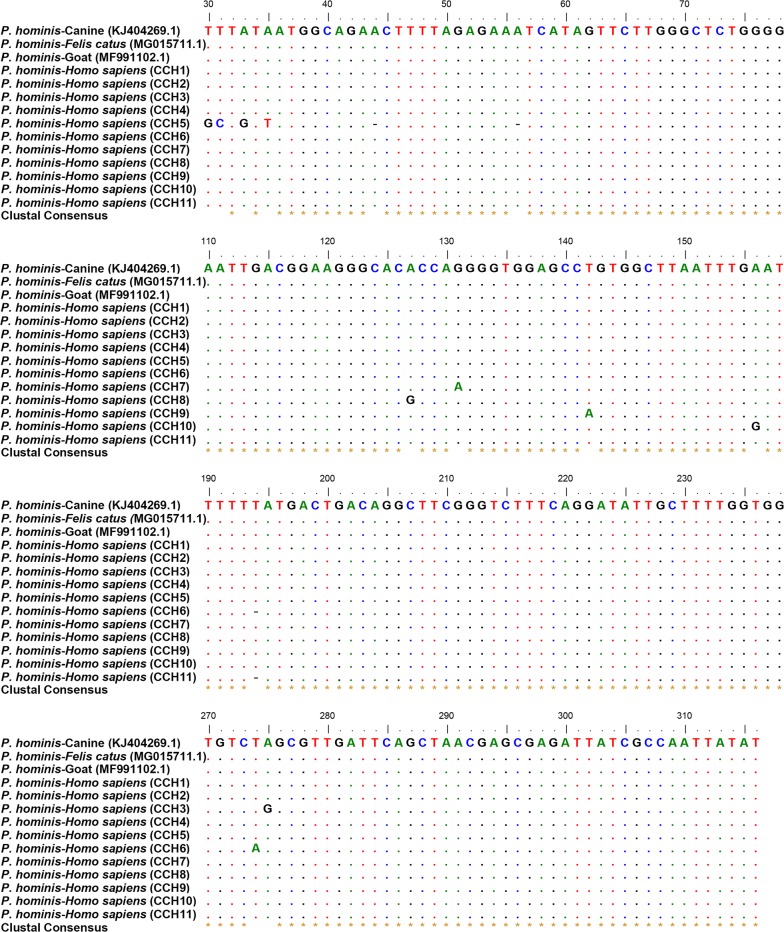
Alignment of the sequences of partial *18S* rRNA gene of *P. hominis* isolated from humans in the present study with reference sequences. Nucleotides that differ from those in the reference sequences are indicated. Dots represent the consensus nucleotides in all the sequences. *Abbreviation*: CCH, China Changchun human

**Fig. 3:**
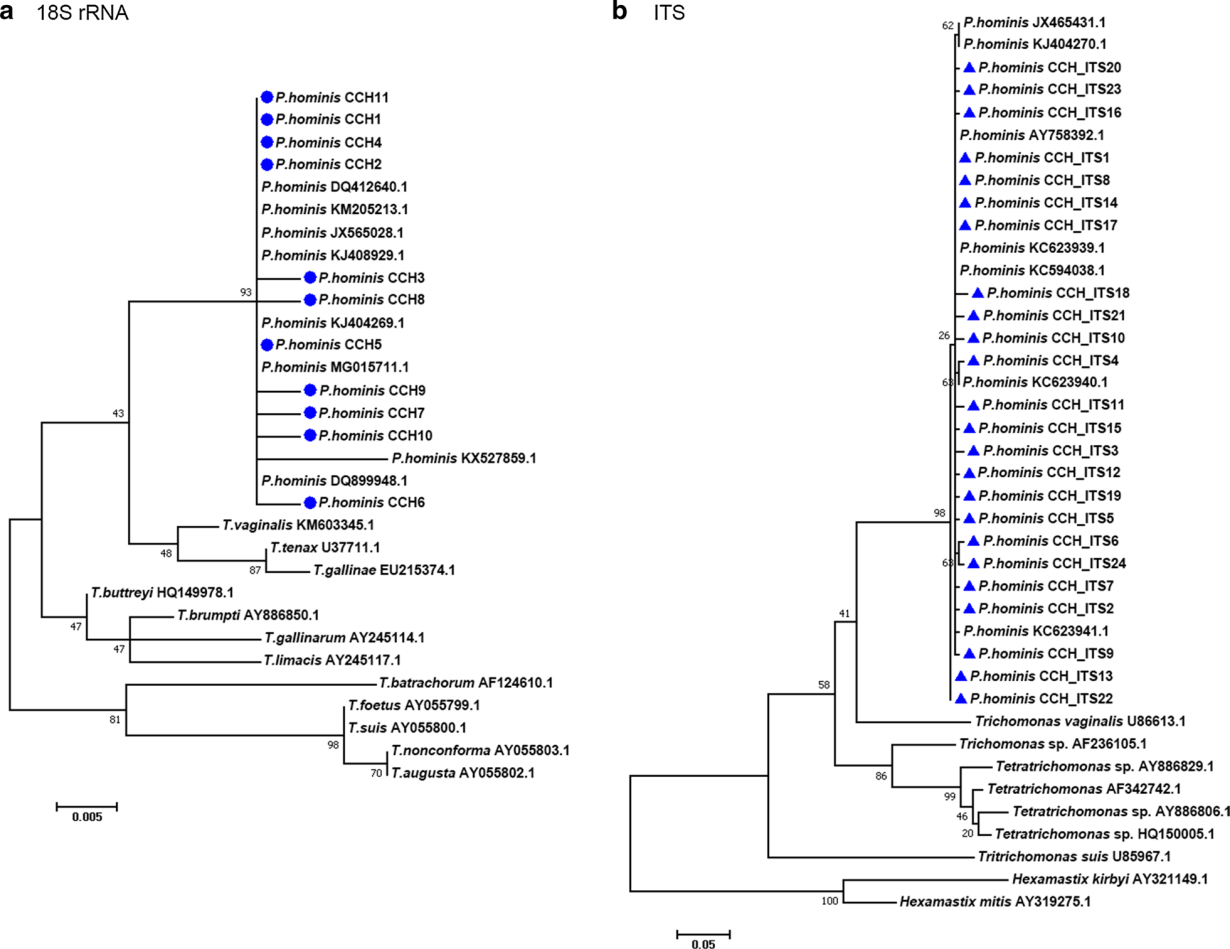
Phylogenetic relationships between *P. hominis* obtained in the present study (marked with blue circle or blue triangle) and other known trichomonads were inferred using the maximum likelihood analysis of the partial *18S* rRNA gene and ITS sequence based on the genetic distance calculated by Kimura 2-parameter model. The numbers at the branches represent the percentage of replicate trees in the bootstrap test (1000 replicates). **a** *18S* rRNA sequences. **b** ITS sequences. *Abbreviation*: CCH, China Changchun human

BLAST (Basic Local Alignment Search Tool) analyses of the partial *18S* rRNA gene and the ITS region indicated that *P. hominis* in both control population and cancer patients were homologous to the strains isolated from dogs, cats, etc. Of the 94 partial *18S* rRNA sequences obtained, 81 sequences displayed 100% identity to the reference sequence (GenBank: KJ404269; Changchun canine strain). Thirteen sequences were assigned to novel types with a number of single nucleotide polymorphisms (SNPs) compared to KJ404269 (99% identity) including substitutions, insertion and deletion of a single nucleotide (Fig. 2, Table 3). These sequences were classified into 10 types (CCH2–11, Table 3). Additionally, the ITS sequences obtained were homologous to the reference sequence (GenBank: KJ404270.1; Changchun canine strain) with a number of SNPs. These sequences were classified into 24 classes (CCH-ITS1–CCH-ITS24). Finally, the phylogenetic tree demonstrated that all the sequences obtained belonged to *P. hominis* based on the maximum likelihood method (Fig. 3).

**Table 3:** SNPs of the ITS regions of *18S* rRNA gene of *P. hominis* identified in the present study

Group	GenBank ID	Reference sequence	Isolate no.	Type	SNP
Control	MK177542	KJ404269	10	CCH1	–
	MK177543	KJ404269	1	CCH2	A317C
	MK177544	KJ404269	1	CCH3	A275G
	MK177545	KJ404269	1	CCH4	331–332 T insert
Gastrointestinal cancer	MK177542	KJ404269	71	CCH1	–
	MK177546	KJ404269	1	CCH5	T30G; T31C; A33G; A35T; 43 A missing; 54 A missing; 317 A missing
	MK177547	KJ404269	2	CCH6	189 T missing; T274A
	MK177548	KJ404269	2	CCH7	G131A
	MK177549	KJ404269	1	CCH8	A127 G
	MK177550	KJ404269	1	CCH9	T142A
	MK177551	KJ404269	1	CCH10	A156G
	MK177552	KJ404269	1	CCH11	189 T missing; 317 A missing
	MK177545	KJ404269	1	CCH4	331–332 T insert

*Abbreviation*: CCH, China Changchun human; SNP, Single nucleotide polymorphisms

## Discussion

*Pentatrichomonas hominis* is an anaerobic and flagellated protozoan that infects a wide range of hosts including humans [23, 27]. In a previous study [27], the prevalence of *P. hominis* infection in dogs, humans and monkeys was found to be 27.38%, 4.00% and 46.67%, respectively. Currently, *P. hominis* is considered to be a potential zoonotic parasite and can cause several symptoms, such as diarrhea [31, 32]. However, the study of the association between *P. hominis* infection and cancer is still lacking. To our knowledge, the present study is the first report on the prevalence and polymorphism of *P. hominis* in patients with gastrointestinal cancers. *Pentatrichomonas hominis* was found to be significantly associated with gastrointestinal cancers. Typically, *P. hominis* infection was highly associated with colorectal cancer (OR: 5.93; 95% CI: 3.00–11.77) and stomach cancer (OR: 7.11; 95% CI: 3.13–16.11), indicating that *P. hominis* infection could be a potential risk factor for these cancers.

*Pentatrichomonas hominis* belongs to Trichomonadida which are often morphologically recognized by the presence of three to five anterior flagella and a single recurrent flagellum [36]. Trichomonadids are composed of a large group of common protozoans including both pathogenic and commensal species. Among them, only four species are considered as human parasites, including *T. vaginalis*, *Trichomonas tenax*, *P. hominis* and *Dientamoeba fragilis*. The first species is human-specific, while the other three are potentially zoonotic [31, 37]. A recent study demonstrated that *T. musculis*, a murine trichomonadid, can exacerbate colorectal cancer development through induction of IL-18 in mice [18]. This study also showed that *D. fragilis* is the closest human ortholog of *T. musculis*, suggesting it may also play a role in cancer progression [18]. In addition, *T. vaginalis* was shown to be associated with cervical and prostate cancers [16, 17]. Thus, the pathological role of *P. hominis* in cancer is speculated. Indeed, we here identified a high association between *P. hominis* infections and gastrointestinal cancers. *Pentatrichomonas hominis* infection can cause a 6.75-fold (95% CI: 3.55–12.83) risk of gastrointestinal cancers. The high prevalence of *P. hominis* was not only observed in colorectal cancer, but also in other gastrointestinal cancers, including stomach cancer, liver cancer, esophageal cancer and small intestine cancer.

It is well-known that *P. hominis* mainly colonizes the large intestine in humans [23, 27]. How *P. hominis* infections influence other gastrointestinal cancers is elusive. Although unlikely, it is possible that *P. hominis* inhabit the atypical locations, such as liver and small intestine. In support of this speculation, *P. hominis* has been rarely identified in the respiratory tract [33], suggesting that *P. hominis* can grow outside of its usual locations. Alternatively, *P. hominis* infections may alter the microbiota of the gastrointestinal tract, which in turn affects these cancers. It has been shown that microbiota are highly associated with gastrointestinal and non-gastrointestinal cancers, such as colorectal cancer, liver cancer and breast cancer [38–43]. Meanwhile, infections with several parasites can perturb the diversity and relative abundance of intestinal microbiota. For instance, persistent infection with *Trichuris muris*, a parasite localized in the large intestine, dramatically decreased the diversity of bacterial communities, while increasing the relative abundance of the genus *Lactobacillus* in the murine intestine [44]. *Cryptosporidium parvum* infections also perturbed the composition of intestinal microbiota in mice [45]. In addition, *Blastocystis* spp. alone or in co-infections with *D. fragilis* decreased the relative abundance of *Bacteroides* and Clostridial cluster XIVa and increased the levels of *Prevotella* [46]. Future studies are warranted to investigate how *P. hominis* infections could potentially influence, or alternatively be favored by, different gastrointestinal cancers.

A variety of species have been identified as hosts of *P. hominis*, including humans, dogs, pigs, monkeys, cats, sheep and cattle [23–27, 47]. In the present study, sequence analysis revealed that 81 partial *18S* rRNA sequences obtained were 100% identical to that of *P. hominis* Changchun canine strain (GenBank: KJ404269) isolated from a dog [48], and 13 novel sequences displayed nucleotide variations compared with KJ404269. We further identified that the sequence KJ404269 is identical with MG015711 and MF991102 isolated from cats and goats, respectively (Fig. 2) [25, 49]. Additionally, the ITS sequences obtained were homologous to the reference strain KJ404270.1 isolated from a dog [48]. Therefore, we speculate that cancer patients might acquire *P. hominis* infections from the fecal material of dogs, cats or goats. Furthermore, a phylogenetic analysis clearly showed that partial *18S* rRNA and ITS sequences were genetically clustered with known *P. hominis* sequences isolated from dogs, cats, goats, etc, suggesting potential zoonotic transmission.

## Conclusions

This case-control study represents the first report of the association between *P. hominis* infections and gastrointestinal cancers. It evokes the reconsideration of the pathogenic role of *P. hominis* in human health. The polymorphism of *P. hominis* identified in this study supports potential zoonotic transmission. Further work is required to elucidate the pathological function of *P. hominis* in cancer induction and/or progression.

## Supplementary information

**Additional file 1: Table S1.** Information on the sex, age, residence, cancer subtype and *P. hominis* infection for 195 gastrointestinal cancer patients.

**Additional file 2: Table S2.** Information on the sex, age, residence and *P. hominis* infection for the control population.

**Additional file 3: Table S3.** Prevalence of *P. hominis* infections in the control population by selected characteristics.

**Additional file 4: Table S4.** Prevalence of *P. hominis* infections in stomach cancer patients by selected characteristics.

## Data Availability

The data generated and analyzed during this study are included within this article and its additional files. The newly generated sequences were submitted to the GenBank database under the Accession Numbers MK177542–MK177552, MN173974–MN173996 and MN189982.

## Abbreviations

CI: confidence interval
ITS: internal transcribed spacer
OR: odds ratio
SNP: single nucleotide polymorphisms
