# Supplementary material for: High prevalence of Pentatrichomonas hominis infection in gastrointestinal cancer patients
Source: Parasit Vectors. 2019 Aug 28;12:423. doi: 10.1186/s13071-019-3684-4 (PMC6714378; doi:10.1186/s13071-019-3684-4)
Supplement: Supplementary file 1 — Additional file 1: Table S1. Information on the sex, age, residence, cancer subtype and P. hominis infection for 195 gastrointestinal cancer patients. [file 13071_2019_3684_MOESM1_ESM.docx]

**Additional file 1: Table S1. Information on the sex, age, residence, cancer subtype and *P. hominis* infection for 195 gastrointestinal cancer patients.**

|  | **Sex** | **Age** | **Residence** | **Cancer types** | ***P. hominis* infection** |
| --- | --- | --- | --- | --- | --- |
| 1 | Male | 65 | Urban | Colorectal cancer | Positive |
| 2 | Male | 52 | Urban | Colorectal cancer | Positive |
| 3 | Male | 36 | Urban | Colorectal cancer | Positive |
| 4 | Male | 62 | Rural | Colorectal cancer | Positive |
| 5 | Male | 63 | Urban | Colorectal cancer | Positive |
| 6 | Male | 46 | Urban | Colorectal cancer | Positive |
| 7 | Male | 54 | Rural | Colorectal cancer | Positive |
| 8 | Male | 65 | Urban | Colorectal cancer | Positive |
| 9 | Male | 50 | Urban | Colorectal cancer | Positive |
| 10 | Male | 51 | Urban | Colorectal cancer | Positive |
| 11 | Male | 57 | Urban | Colorectal cancer | Positive |
| 12 | Male | 57 | Rural | Colorectal cancer | Positive |
| 13 | Male | 66 | Rural | Colorectal cancer | Positive |
| 14 | Male | 80 | Rural | Colorectal cancer | Positive |
| 15 | Male | 55 | Urban | Colorectal cancer | Positive |
| 16 | Male | 58 | Urban | Colorectal cancer | Positive |
| 17 | Male | 40 | Urban | Colorectal cancer | Positive |
| 18 | Male | 54 | Rural | Colorectal cancer | Positive |
| 19 | Male | 59 | Urban | Colorectal cancer | Positive |
| 20 | Male | 66 | Rural | Colorectal cancer | Positive |
| 21 | Male | 68 | Urban | Colorectal cancer | Positive |
| 22 | Male | 33 | Urban | Colorectal cancer | Positive |
| 23 | Male | 57 | Rural | Colorectal cancer | Positive |
| 24 | Male | 61 | Rural | Colorectal cancer | Positive |
| 25 | Male | 52 | Urban | Colorectal cancer | Positive |
| 26 | Female | 56 | Urban | Colorectal cancer | Positive |
| 27 | Female | 36 | Rural | Colorectal cancer | Positive |
| 28 | Female | 63 | Urban | Colorectal cancer | Positive |
| 29 | Female | 68 | Urban | Colorectal cancer | Positive |
| 30 | Female | 66 | Urban | Colorectal cancer | Positive |
| 31 | Female | 50 | Rural | Colorectal cancer | Positive |
| 32 | Female | 62 | Urban | Colorectal cancer | Positive |
| 33 | Female | 63 | Urban | Colorectal cancer | Positive |
| 34 | Female | 61 | Rural | Colorectal cancer | Positive |
| 35 | Female | 61 | Rural | Colorectal cancer | Positive |
| 36 | Female | 60 | Urban | Colorectal cancer | Positive |
| 37 | Female | 48 | Urban | Colorectal cancer | Positive |
| 38 | Female | 65 | Rural | Colorectal cancer | Positive |
| 39 | Female | 59 | Rural | Colorectal cancer | Positive |
| 40 | Female | 47 | Urban | Colorectal cancer | Positive |
| 41 | Female | 66 | Urban | Colorectal cancer | Positive |
| 42 | Female | 69 | Urban | Colorectal cancer | Positive |
| 43 | Female | 65 | Urban | Colorectal cancer | Positive |
| 44 | Female | 48 | Urban | Colorectal cancer | Positive |
| 45 | Male | 56 | Rural | Colorectal cancer | Negative |
| 46 | Male | 62 | Rural | Colorectal cancer | Negative |
| 47 | Male | 68 | Rural | Colorectal cancer | Negative |
| 48 | Male | 64 | Rural | Colorectal cancer | Negative |
| 49 | Male | 50 | Rural | Colorectal cancer | Negative |
| 50 | Male | 60 | Urban | Colorectal cancer | Negative |
| 51 | Male | 68 | Rural | Colorectal cancer | Negative |
| 52 | Male | 61 | Rural | Colorectal cancer | Negative |
| 53 | Male | 61 | Rural | Colorectal cancer | Negative |
| 54 | Male | 79 | Urban | Colorectal cancer | Negative |
| 55 | Male | 48 | Urban | Colorectal cancer | Negative |
| 56 | Male | 49 | Urban | Colorectal cancer | Negative |
| 57 | Male | 65 | Rural | Colorectal cancer | Negative |
| 58 | Male | 59 | Urban | Colorectal cancer | Negative |
| 59 | Male | 61 | Rural | Colorectal cancer | Negative |
| 60 | Male | 61 | Urban | Colorectal cancer | Negative |
| 61 | Male | 53 | Rural | Colorectal cancer | Negative |
| 62 | Male | 78 | Urban | Colorectal cancer | Negative |
| 63 | Male | 53 | Urban | Colorectal cancer | Negative |
| 64 | Male | 45 | Urban | Colorectal cancer | Negative |
| 65 | Male | 48 | Urban | Colorectal cancer | Negative |
| 66 | Male | 46 | Rural | Colorectal cancer | Negative |
| 67 | Male | 66 | Urban | Colorectal cancer | Negative |
| 68 | Male | 50 | Rural | Colorectal cancer | Negative |
| 69 | Male | 61 | Urban | Colorectal cancer | Negative |
| 70 | Male | 43 | Urban | Colorectal cancer | Negative |
| 71 | Male | 55 | Urban | Colorectal cancer | Negative |
| 72 | Male | 63 | Urban | Colorectal cancer | Negative |
| 73 | Male | 69 | Urban | Colorectal cancer | Negative |
| 74 | Male | 58 | Rural | Colorectal cancer | Negative |
| 75 | Male | 63 | Rural | Colorectal cancer | Negative |
| 76 | Male | 54 | Urban | Colorectal cancer | Negative |
| 77 | Male | 74 | Urban | Colorectal cancer | Negative |
| 78 | Male | 63 | Urban | Colorectal cancer | Negative |
| 79 | Male | 70 | Urban | Colorectal cancer | Negative |
| 80 | Male | 59 | Urban | Colorectal cancer | Negative |
| 81 | Male | 60 | Urban | Colorectal cancer | Negative |
| 82 | Male | 68 | Urban | Colorectal cancer | Negative |
| 83 | Male | 59 | Rural | Colorectal cancer | Negative |
| 84 | Male | 35 | Urban | Colorectal cancer | Negative |
| 85 | Male | 45 | Rural | Colorectal cancer | Negative |
| 86 | Male | 52 | Rural | Colorectal cancer | Negative |
| 87 | Female | 48 | Rural | Colorectal cancer | Negative |
| 88 | Female | 48 | Urban | Colorectal cancer | Negative |
| 89 | Female | 53 | Urban | Colorectal cancer | Negative |
| 90 | Female | 67 | Urban | Colorectal cancer | Negative |
| 91 | Female | 63 | Urban | Colorectal cancer | Negative |
| 92 | Female | 48 | Urban | Colorectal cancer | Negative |
| 93 | Female | 41 | Rural | Colorectal cancer | Negative |
| 94 | Female | 59 | Urban | Colorectal cancer | Negative |
| 95 | Female | 69 | Rural | Colorectal cancer | Negative |
| 96 | Female | 74 | Urban | Colorectal cancer | Negative |
| 97 | Female | 51 | Urban | Colorectal cancer | Negative |
| 98 | Female | 63 | Urban | Colorectal cancer | Negative |
| 99 | Female | 44 | Rural | Colorectal cancer | Negative |
| 100 | Female | 48 | Rural | Colorectal cancer | Negative |
| 101 | Female | 52 | Urban | Colorectal cancer | Negative |
| 102 | Female | 64 | Urban | Colorectal cancer | Negative |
| 103 | Female | 70 | Rural | Colorectal cancer | Negative |
| 104 | Female | 64 | Urban | Colorectal cancer | Negative |
| 105 | Female | 45 | Rural | Colorectal cancer | Negative |
| 106 | Female | 65 | Urban | Colorectal cancer | Negative |
| 107 | Female | 66 | Urban | Colorectal cancer | Negative |
| 108 | Female | 63 | Urban | Colorectal cancer | Negative |
| 109 | Female | 68 | Urban | Colorectal cancer | Negative |
| 110 | Female | 47 | Rural | Colorectal cancer | Negative |
| 111 | Female | 53 | Rural | Colorectal cancer | Negative |
| 112 | Female | 83 | Rural | Colorectal cancer | Negative |
| 113 | Female | 65 | Rural | Colorectal cancer | Negative |
| 114 | Female | 70 | Urban | Colorectal cancer | Negative |
| 115 | Female | 74 | Urban | Colorectal cancer | Negative |
| 116 | Female | 61 | Urban | Colorectal cancer | Negative |
| 117 | Male | 56 | Urban | Stomach cancer | Positive |
| 118 | Male | 75 | Rural | Stomach cancer | Positive |
| 119 | Male | 48 | Rural | Stomach cancer | Positive |
| 120 | Male | 57 | Rural | Stomach cancer | Positive |
| 121 | Male | 59 | Rural | Stomach cancer | Positive |
| 122 | Male | 53 | Rural | Stomach cancer | Positive |
| 123 | Male | 78 | Urban | Stomach cancer | Positive |
| 124 | Male | 64 | Rural | Stomach cancer | Positive |
| 125 | Male | 60 | Urban | Stomach cancer | Positive |
| 126 | Male | 72 | Urban | Stomach cancer | Positive |
| 127 | Male | 62 | Rural | Stomach cancer | Positive |
| 128 | Male | 43 | Urban | Stomach cancer | Positive |
| 129 | Male | 57 | Urban | Stomach cancer | Positive |
| 130 | Male | 64 | Rural | Stomach cancer | Positive |
| 131 | Male | 68 | Urban | Stomach cancer | Positive |
| 132 | Male | 73 | Rural | Stomach cancer | Positive |
| 133 | Male | 55 | Urban | Stomach cancer | Positive |
| 134 | Male | 61 | Urban | Stomach cancer | Positive |
| 135 | Male | 67 | Rural | Stomach cancer | Positive |
| 136 | Male | 55 | Rural | Stomach cancer | Positive |
| 137 | Male | 79 | Urban | Stomach cancer | Positive |
| 138 | Female | 44 | Urban | Stomach cancer | Positive |
| 139 | Female | 54 | Rural | Stomach cancer | Positive |
| 140 | Male | 49 | Urban | Stomach cancer | Negative |
| 141 | Male | 60 | Rural | Stomach cancer | Negative |
| 142 | Male | 61 | Urban | Stomach cancer | Negative |
| 143 | Male | 60 | Rural | Stomach cancer | Negative |
| 144 | Male | 65 | Rural | Stomach cancer | Negative |
| 145 | Male | 60 | Rural | Stomach cancer | Negative |
| 146 | Male | 63 | Rural | Stomach cancer | Negative |
| 147 | Male | 31 | Urban | Stomach cancer | Negative |
| 148 | Male | 40 | Rural | Stomach cancer | Negative |
| 149 | Male | 45 | Rural | Stomach cancer | Negative |
| 150 | Male | 68 | Urban | Stomach cancer | Negative |
| 151 | Male | 63 | Urban | Stomach cancer | Negative |
| 152 | Male | 38 | Rural | Stomach cancer | Negative |
| 153 | Male | 58 | Urban | Stomach cancer | Negative |
| 154 | Male | 64 | Rural | Stomach cancer | Negative |
| 155 | Male | 58 | Urban | Stomach cancer | Negative |
| 156 | Male | 66 | Rural | Stomach cancer | Negative |
| 157 | Female | 58 | Urban | Stomach cancer | Negative |
| 158 | Female | 53 | Urban | Stomach cancer | Negative |
| 159 | Female | 75 | Urban | Stomach cancer | Negative |
| 160 | Female | 87 | Urban | Stomach cancer | Negative |
| 161 | Female | 45 | Rural | Stomach cancer | Negative |
| 162 | Female | 42 | Rural | Stomach cancer | Negative |
| 163 | Female | 54 | Rural | Stomach cancer | Negative |
| 164 | Female | 58 | Urban | Stomach cancer | Negative |
| 165 | Female | 56 | Urban | Stomach cancer | Negative |
| 166 | Female | 80 | Rural | Stomach cancer | Negative |
| 167 | Female | 42 | Urban | Stomach cancer | Negative |
| 168 | Male | 68 | Rural | Esophageal cancer | Positive |
| 169 | Male | 55 | Rural | Esophageal cancer | Positive |
| 170 | Male | 55 | Rural | Esophageal cancer | Positive |
| 171 | Male | 61 | Urban | Esophageal cancer | Positive |
| 172 | Male | 46 | Urban | Esophageal cancer | Positive |
| 173 | Male | 59 | Urban | Esophageal cancer | Positive |
| 174 | Female | 63 | Rural | Esophageal cancer | Positive |
| 175 | Male | 49 | Rural | Esophageal cancer | Negative |
| 176 | Male | 66 | Rural | Esophageal cancer | Negative |
| 177 | Male | 66 | Urban | Esophageal cancer | Negative |
| 178 | Male | 60 | Urban | Esophageal cancer | Negative |
| 179 | Male | 64 | Urban | Esophageal cancer | Negative |
| 180 | Male | 69 | Urban | Esophageal cancer | Negative |
| 181 | Male | 58 | Rural | Esophageal cancer | Negative |
| 182 | Female | 63 | Rural | Esophageal cancer | Negative |
| 183 | Female | 55 | Rural | Esophageal cancer | Negative |
| 184 | Male | 52 | Urban | Liver cancer | Positive |
| 185 | Male | 42 | Urban | Liver cancer | Positive |
| 186 | Male | 82 | Urban | Liver cancer | Positive |
| 187 | Female | 45 | Rural | Liver cancer | Positive |
| 188 | Male | 55 | Urban | Liver cancer | Negative |
| 189 | Male | 49 | Rural | Liver cancer | Negative |
| 190 | Female | 63 | Rural | Liver cancer | Negative |
| 191 | Male | 55 | Urban | Small intestinal cancer | Positive |
| 192 | Female | 63 | Urban | Small intestinal cancer | Positive |
| 193 | Female | 65 | Urban | Small intestinal cancer | Positive |
| 194 | Male | 58 | Urban | Small intestinal cancer | Negative |
| 195 | Female | 58 | Urban | Small intestinal cancer | Negative |
