# Supplementary material for: High prevalence of Pentatrichomonas hominis infection in gastrointestinal cancer patients
Source: Parasit Vectors. 2019 Aug 28;12:423. doi: 10.1186/s13071-019-3684-4 (PMC6714378; doi:10.1186/s13071-019-3684-4)
Supplement: Supplementary file 3 — Additional file 3: Table S3. Prevalence of P. hominis infections in the control population by selected characteristics. [file 13071_2019_3684_MOESM3_ESM.docx]

**Additional file 3: Table S3. Prevalence of *P. hominis* infections in the control population by selected characteristics**

| **Group** | **Control population (*n*=142)** | | | |
| --- | --- | --- | --- | --- |
|  | **No. examined** | **No. positive (%)** | **No. negative (%)** | ***χ*^2^/*df*/*P*-value** |
| Age (years) |  |  |  |  |
| <50 | 22 | 3 (13.64) | 19 (86.36) | 1.08/0.56^a^ |
| 50-60 | 41 | 4 (9.76) | 37 (90.24) |  |
| >60 | 79 | 6 (7.59) | 73 (92.41) |  |
| Sex |  |  |  |  |
| Male | 71 | 8 (11.27) | 63 (88.73) | 0.76/1/0.38 |
| Female | 71 | 5 (7.04) | 66 (92.96) |  |
| Residence^b^ |  |  |  |  |
| Urban | 90 | 9 (10) | 81 (90) | 0.02/1/0.9 |
| Rural | 51 | 4 (7.84) | 47 (92.16) |  |

^a^ Fisher’s exact test.

**^b^** One case was excluded due to lack of selected information.
