# Supplementary material for: High prevalence of Pentatrichomonas hominis infection in gastrointestinal cancer patients
Source: Parasit Vectors. 2019 Aug 28;12:423. doi: 10.1186/s13071-019-3684-4 (PMC6714378; doi:10.1186/s13071-019-3684-4)
Supplement: Supplementary file 4 — Additional file 4: Table S4. Prevalence of P. hominis infections in stomach cancer patients by selected characteristics. [file 13071_2019_3684_MOESM4_ESM.docx]

**Additional file 4: Table S4. Prevalence of *P. hominis* infections in stomach cancer patients by selected characteristics**

| **Group** | **Stomach cancer (*n*=51)** | | | |
| --- | --- | --- | --- | --- |
|  | **No. examined** | **No. positive (%)** | **No. negative (%)** | ***χ*^2^/*df*/*P*-value** |
| Age (years) |  |  |  |  |
| <50 | 11 | 3 (27.27) | 8 (72.73) | 1.90/2/0.39 |
| 50-60 | 19 | 9 (47.39) | 10 (52.63) |  |
| >60 | 21 | 11 (52.38) | 10 (47.62) |  |
| Sex |  |  |  |  |
| Male | 38 | 21 (55.26) | 17 (44.74) | 6.22/1/0.01 |
| Female | 13 | 2 (15.38) | 11 (84.62) |  |
| Residence |  |  |  |  |
| Urban | 26 | 12 (46.15) | 14 (53.85) | 0.02/1/0.88 |
| Rural | 25 | 11 (44) | 14 (56) |  |
